# Cardiovascular Efficacy and Safety of Finerenone: A Meta‐Analysis of Randomized Controlled Trials

**DOI:** 10.1002/clc.70065

**Published:** 2025-02-06

**Authors:** Mushood Ahmed, Areeba Ahsan, Aimen Shafiq, Muhammad Talha Maniya, Hritvik Jain, Javed Iqbal, Muhammad Abdullah Naveed, Raheel Ahmed, Jamal S. Rana, Marat Fudim, Gregg C. Fonarow

**Affiliations:** ^1^ Rawalpindi Medical University Rawalpindi Pakistan; ^2^ Foundation University Medical College Islamabad Pakistan; ^3^ Dow University of Health Sciences Karachi Pakistan; ^4^ Ziauddin University Karachi Pakistan; ^5^ All India Institute of Medical Sciences (AIIMS) Jodhpur India; ^6^ Hamad Medical Corporation Doha Qatar; ^7^ Department of Cardiology Royal Brompton Hospital London UK; ^8^ National Heart and Lung Institute, Imperial College London UK; ^9^ Division of Cardiology Kaiser Permanente Northern California Oakland California USA; ^10^ Division of Research Kaiser Permanente Northern California Oakland California USA; ^11^ Department of Medicine Duke University Medical Center Durham North Carolina USA; ^12^ Duke Clinical Research Institute Durham North Carolina USA; ^13^ Division of Cardiology Ahmanson‐UCLA Cardiomyopathy Center Los Angeles California USA

**Keywords:** cardiovascular, chronic kidney disease, finerenone, heart failure

## Abstract

**Background:**

Finerenone, a nonsteroidal mineralocorticoid receptor antagonist, has emerged as a novel therapeutic option for the management of patients with diabetes, chronic kidney disease, or heart failure. We seek to summarize the evidence on the drug's effectiveness regarding cardiovascular (CV) outcomes.

**Methods:**

We conducted a literature search of Pubmed, Cochrane CENTRAL, Embase, and ClinicalTrials.gov from inception to September 2024. Trials exploring the effects of finerenone on CV outcomes were extracted and analyzed. The results of pooled analyses were presented as risk ratios (RRs) with 95% confidence intervals (CIs).

**Results:**

A total of eight trials, incorporating 21 200 patients, were included. The pooled analysis demonstrated a significant reduction in all‐cause death (RR 0.92, 95% CI: 0.85–0.99), major adverse CV events (RR 0.85, 95% CI: 0.81–0.90), heart failure‐related hospitalizations or unplanned hospital visits (RR 0.82, 95% CI: 0.76–0.87) with finerenone administration compared to control. Finerenone use was associated with a trend of reduced risk of CV death without reaching statistical significance (RR 0.90, 95% CI: 0.81–1.00). The risk of myocardial infarction (RR 0.91, 95% CI: 0.74–1.12), adverse events (RR 0.96, 95% CI: 0.89–1.03), adverse events leading to discontinuation (RR 1.06, 95% CI: 0.96–1.17) remained comparable across both groups. However, an increased risk of hyperkalemia (RR 2.07, 95% CI: 1.88–2.27) was observed with finerenone therapy compared to the control group.

**Conclusion:**

Finerenone administration was associated with improved CV outcomes in the CV‐renmetabolic conditions compared to the control group.

## Graphical abstract

This meta‐analysis of 21 200 patients found that finerenone can improve cardiovascular outcomes in patients with diabetes, chronic kidney disease, or heart failure.

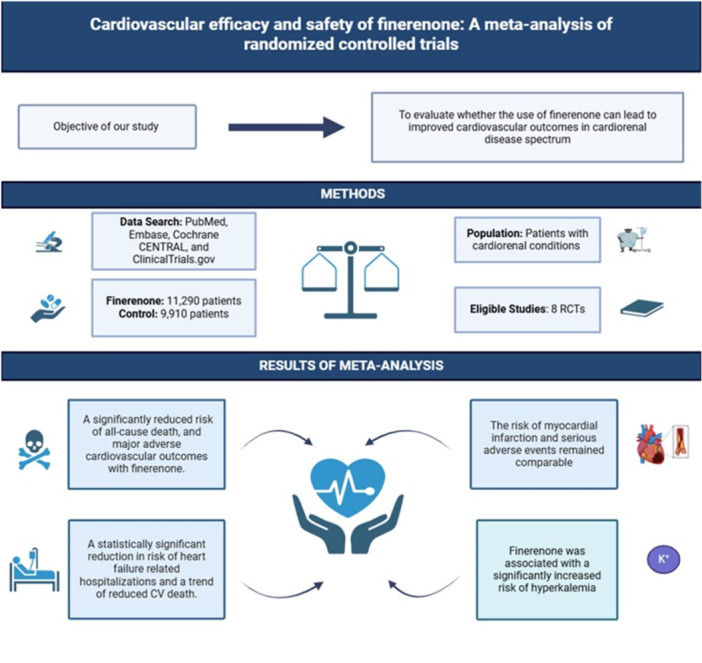

## 1 Introduction

Cardiovascular (CV) disease remains a leading cause of morbidity and death worldwide [1]. In high‐risk individuals, new treatment options for improving CV outcomes have been made possible by recent developments in mineralocorticoid receptor antagonists (MRAs). The mineralocorticoid receptor belongs to the family of nuclear hormone receptors [2]. By inhibiting mineralocorticoid receptors in CV cells, such as cardiomyocytes or fibroblasts, as well as in cells of the innate and adaptive immune system, strong anti‐hypertrophic, anti‐fibrotic, and anti‐inflammatory effects are generated which enhances heart function [3]. And this may translate into improved CV outcomes.

Finerenone is a newer nonsteroidal MRA that has emerged as a novel therapeutic option [4]. While multiple studies have highlighted the potential benefits of finerenone, most of the previous research has focused on populations with chronic kidney disease or diabetes [5, 6, 7, 8] and CV outcomes in different patient populations remain underexplored.

The FINEARTS‐HF trial [9] the first randomized controlled trial (RCT) focuses solely on CV outcomes in heart failure (HF) patients. Hence, it offers a new insight into finerenone's efficacy beyond its traditional role in renal protection. These new data provide a unique opportunity to revisit the role of finerenone, not just as a kidney‐protective agent but as an important drug in managing CV outcomes. Our meta‐analysis seeks to provide an updated and comprehensive evaluation of finerenone's CV efficacy and safety profile.

## 2 Methods

This systematic review and meta‐analysis was carried out following the recommendations outlined in the PRISMA (Preferred Reporting Items for Systematic Reviews and Meta‐Analyses) framework [10]. The protocol of review was registered with PROSPERO (CRD42024585743).

### 2.1 Search Strategy and Data Sources

A comprehensive search was carried out by two independent investigators (A.A. and A.S.), who screened several large databases, including PubMed/MEDLINE, Embase, Cochrane Central Register of Controlled Trials, and ClinicalTrials.gov, covering all studies from inception to September 1, 2024. In addition to the electronic search, manual checks of reference lists from key RCTs, observational studies, prior reviews, and meta‐analyses were conducted to avoid missing any crucial studies. This process involved an extensive search strategy incorporating both text terms and Medical Subject Headings (MeSH) such as (“finerenone,” “cardiovascular outcomes,” “chronic kidney disease,” “diabetes,” and “randomized controlled trial”). The search strings used for each database are provided in Supporting Infomation S1: Table S1.

### 2.2 Inclusion and Exclusion Criteria

To be included in this systematic review and meta‐analysis, studies had to meet predetermined criteria: (i) RCTs assessing the effects of finerenone on CV outcomes; (ii) compared finerenone with placebo or other standard treatments; and (iii) the studies must report at least one of the following key clinical outcomes: all‐cause death, death from CV causes, major adverse cardiovascular events (MACEs) or composite CV endpoint, myocardial infarction (MI), any adverse event (AE), any AE leading to discontinuation of drug, hyperkalemia, and HF‐related hospitalizations (HHF) or unplanned hospital visits. The criteria used for reporting MACE or composite CV endpoint in each trial is reported in Table 1.

**Table 1:** Baseline characteristics of included studies and patients.

Author	Year	Sample size		Composite CV endpoint/MACE	Follow‐up	Intervention group	Control group	Patient population	Age‐ mean SD		males‐ n (%)		BMI‐ mean ± SD		Systolic blood pressure (mean ± SD)		Hypertension, *n* (%)	
		Finerenone	placebo						Finerenone	Placebo	Finerenone	Placebo	Finerenone	Placebo	Finerenone	Placebo	Finerenone	Placebo
Pitt [11]	2021	3686	3666	CV death, MI, stroke, or hospitalization for heart failure	3.4 years	Finerenone 2.5–20 mg once daily	Placebo	T2DM, CKD	64.1 ± 9.7	64.1 ± 10.0	2528 (68.6)	2577 (70.3)	31.5 ± 6.0	31.4 ± 5.9	135.8 ± 14.0	135.7 ± 14.1	3544 (96.1)	3517 (95.9)
Katayama [12]	2017	84	12	—	90 days	Finerenone 1.25–20 mg once daily	Placebo	T2DM, CKD	62.40 ± 9.8	66.75 ± 9.02	67 (79.8)	10 (83.3)	27.18 ± 4.15	26.68 ± 3.24	132.74 ± 13.03	135.72 ± 16.90	80 (95.24)	12 (100.0)
Bakris [13]	2020	2833	2841	CV death, nonfatal MI, stroke, or hospitalization for heart failure.	32 months	Finerenone 10 or 20 mg once daily	Placebo	CKD and DM	65.4 ± 8.9	65.7 ± 9.2	1953 (68.9)	2030 (71.5)	31.1 ± 6.0	31.1 ± 6.0	138.1 ± 14.3	138.0 ± 14.4	2737 (96.6)	2768 (97.4)
Bakris [14]	2015	727	94	—	90 days	Finerenone 1.25–20 mg once daily	Placebo	High albuminuria and DM	64.33 ± 9.21	63.26 ± 8.68	570 (78.4)	69 (73.4)	30.37 ± 5.41	32.49 ± 5.27	139.36 ± 14.40	139.9 ± 14.3	685 (94.2)	89 (94.7)
Fillipatos [15]	2016	834	221	Death from any cause, CV hospitalization or emergency presentation for worsening chronic heart failure	90 days	Finerenone 2.5–20 mg once daily	Eplerenone 25 mg once daily	CKD and/or DM and HFrEF	70.8 ± 10.1	72.4 ± 9.9	645 (88.7)	170 (76.9)	NR	NR	119.67 ± 16.4	121 ± 19	617 [74]	158 (71.5)
Sato [16]	2016	59	13	—	90 days	Finerenone 2.5–20 mg once daily	Eplerenone 25 mg once daily	CKD and/or DM and HFrEF	73.1	76.5	41 (56.9)	12 (92.3)	NR	NR	113.6	110.9	41 (69.4)	10 (76.9)
Solomon [9]	2024	3003	2998	Composite of total worsening heart failure events and CV death	32 months	Finerenone 20–40 mg once daily	Placebo	Symptomatic heart failure and LVEF of 40% or greater	71.9 ± 9.6	72.0 ± 9.7	Females: 1355 (45.1)	1377 (45.9)	29.9 ± 6.1	30.0 ± 6.1	129.5 ± 15.3	129.3 ± 15.3	2640 (87.9)	2685 (89.6)
Pitt^a^ [17]	2013	64	65	—	14 days	BAY 94‐8862 (2.5, 5, or 10 mg q.d., or 5 mg twice daily)	Placebo	HFrEF and mild CKD	72.1 [40–89]^b^		312 (79.6)		28.8 (18.1–46.9)^b^		127.3 (81–180)^b^		261 (66.6)	

Abbreviations: BMI, body mass index; CKD, chronic kidney disease; CV, cardiovascular; HFrEF, heart failure with reduced ejection fraction; *n*, number; NR, not reported; T2DM, type 2 diabetes mellitus.

^a^ The study did not report details of baseline characteristics for individual groups.

^b^ The data are reported as median and interquartile range.

Studies were excluded if they were observational studies and focused on outcomes unrelated to CV events. Editorials, reviews and commentaries were also excluded.

### 2.3 Study Selection and Data Extraction

The search results were imported into the reference management software to remove duplicate records. Two researchers (A.A. and A.S.) independently screened the titles and abstracts of the identified studies. After this initial screening, a full‐text review was conducted on articles that appeared to meet the eligibility criteria. Any disagreements between the two reviewers were resolved through discussion, or when necessary, by involving a third senior author (M.A.).

For each included study, data were meticulously extracted using a pre‐piloted data extraction form. Extracted data included the author's name, year of publication, sample size, participant demographics (such as gender distribution and mean age), various comorbidities, trial details, dosage of finerenone, follow‐up period, and the outcomes.

### 2.4 Assessment of Risk of Bias

The quality of each included RCT was assessed using the latest Cochrane Risk of Bias tool version 2.0 (RoB 2.0) [18], which evaluates the methodological soundness across five core domains: (i) the process used to generate the randomization sequence, (ii) any deviations from the intended intervention, (iii) handling of missing data, (iv) accuracy and validity of outcome measurement, and (v) potential selective reporting of results. Each domain was rated as either “low risk,” “some concerns,” or “high risk” of bias. Furthermore, an overall risk of bias judgment was assigned for each study based on the collective assessment of all domains. This assessment process was performed by two independent reviewers and verified by a third reviewer.

### 2.5 Statistical Analysis

Data analysis was performed using R statistical software (version 4.4.1), with the meta‐analysis being conducted through the “meta” and “metasens” packages within RStudio. To calculate pooled estimates, risk ratios (RRs) and 95% confidence intervals (CIs) were generated using a random‐effects model, which was selected to account for anticipated variability between studies [19]. The Mantel–Haenszel method was employed for pooling dichotomous outcomes. And the Paule–Mandel method was used to estimate the between‐study variance (*τ*²) [20]. The extent of heterogeneity between studies was assessed using the *I*² statistic, with thresholds for interpretation as follows: 0%–40% suggesting low heterogeneity, 30%–60% indicating moderate heterogeneity, 50%–90% reflecting substantial heterogeneity, and 75%–100% signifying considerable heterogeneity [21]. In addition, a sensitivity analysis was carried out using the leave‐one‐out approach, where each study was removed one at a time to evaluate the impact on the overall results. This ensured that no single study disproportionately influenced the findings. The threshold for statistical significance was set at a *p*‐value less than 0.05 for all analyses.

## 3 Results

### 3.1 Literature Search Results

A total of 2004 records were identified after an initial literature search. The 447 duplicate articles were excluded and 1557 articles were screened by two independent investigators using their titles and abstracts; 1496 records were removed and full‐texts of 61 studies were retrieved. The review of full texts identified eight eligible RCTs as reported in Supporting Infomation S1: Figure S1.

### 3.2 Study Characteristics and Quality Assessment

This meta‐analysis consisted of 21 200 patients across eight studies [9, 11, 12, 13, 14, 15, 16, 17], with 11 290 patients receiving finerenone and 9910 receiving control. Two of the included studies used eplerenone as a control while the others used placebo. All study arms had more men than women, with a mean age ranging from 62.4 to 73.1 years, and a follow‐up duration ranging from 90 days to 3.4 years. The baseline characteristics of the included studies are summarized in Table 1.

**Figure 1:**
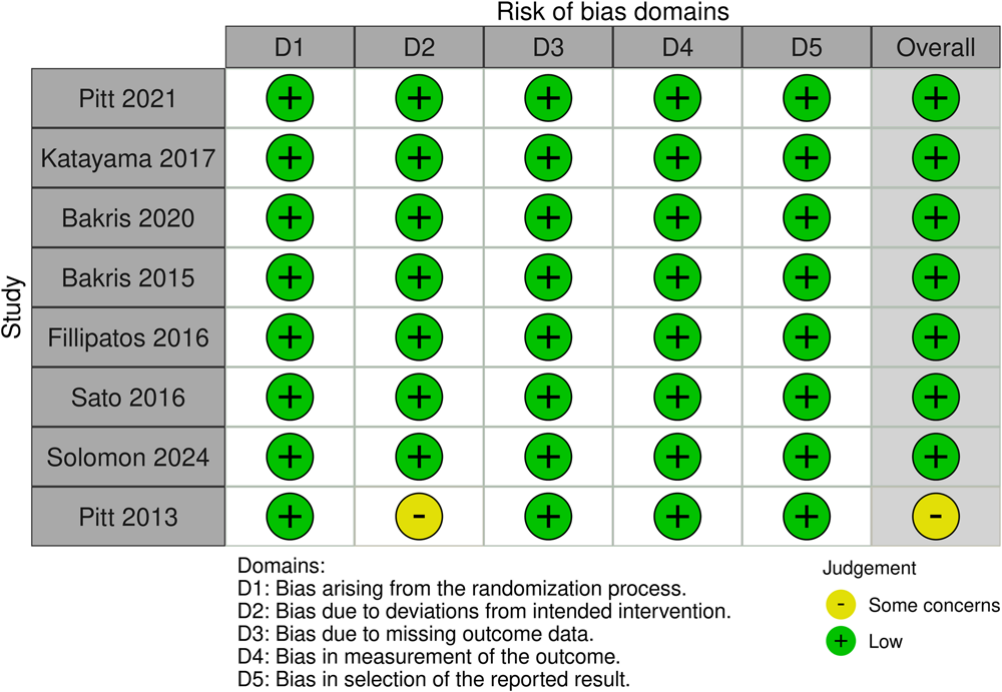
Risk of bias summary in included trials.

The risk of bias assessment revealed that seven included studies were of high methodological quality, exhibiting a low risk of bias in all key domains however some concerns were observed in one trial [17] due to the open‐label design of study. The risk of bias summary is presented in Figure 1.

### 3.3 Results of Meta‐Analysis

#### 3.3.1 All‐Cause Death

**Figure 2:**
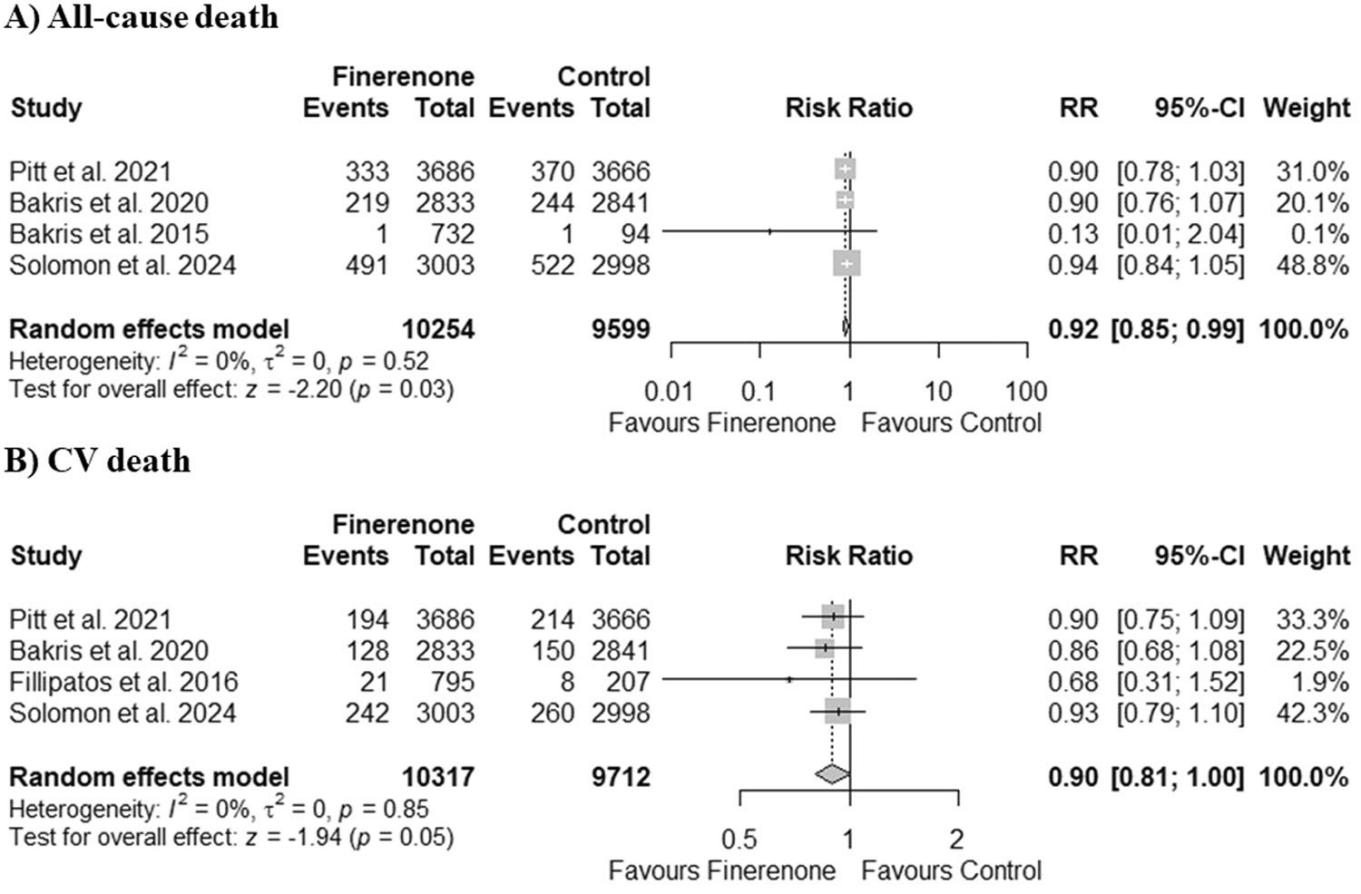
Forest plots for (A) all‐cause death and (B) CV death. CV, cardiovascular.

Four out of eight included studies reported all‐cause death. Our pooled analysis demonstrated a significant reduction in all‐cause death with finerenone therapy compared to the control group (RR 0.92, 95% CI: 0.85–0.99; *p* = 0.03; Figure 2A). There was no heterogeneity between studies (*I* ^2^ = 0%).

#### 3.3.2 CV Death

Four out of eight included studies reported CV death. Our pooled analysis demonstrated a trend of reduced CV death with finerenone therapy compared to the control group (RR 0.90, 95% CI: 0.81–1.00; *p* = 0.05; Figure 2B) without reaching statistical significance. There was no heterogeneity between studies (*I* ^2^ = 0%).

#### 3.3.3 MACEs

**Figure 3:**
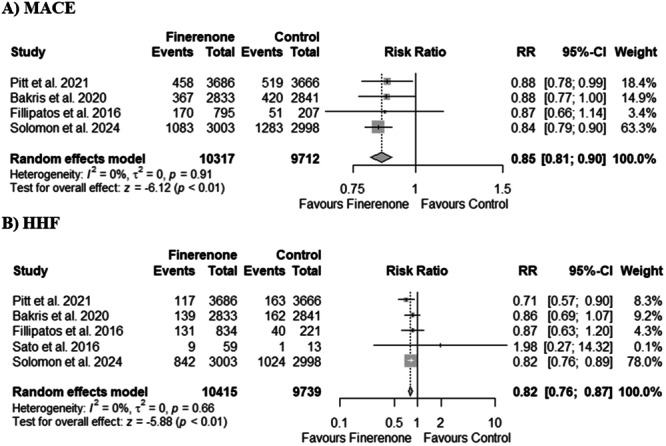
Forest plots for (A) MACE and (B) HHF. HHF, heart failure‐related hospitalizations or unplanned hospital visits.

Four out of eight included studies reported MACE. Our pooled analysis demonstrates a significant reduction in MACE with finerenone therapy compared to the control group (RR 0.85, 95% CI: 0.81–0.90; *p* < 0.01; Figure 3A). There was no heterogeneity between studies (*I* ^2^ = 0%).

#### 3.3.4 HFH

Five out of eight included studies reported HHF or unplanned hospital visits. Our pooled analysis demonstrated a significant reduction in HFH with finerenone therapy compared to the control group (RR 0.82, 95% CI: 0.76–0.87; *p* < 0.01; Figure 3B). There was no heterogeneity between studies (*I* ^2^ = 0%).

#### 3.3.5 MI

**Figure 4:**
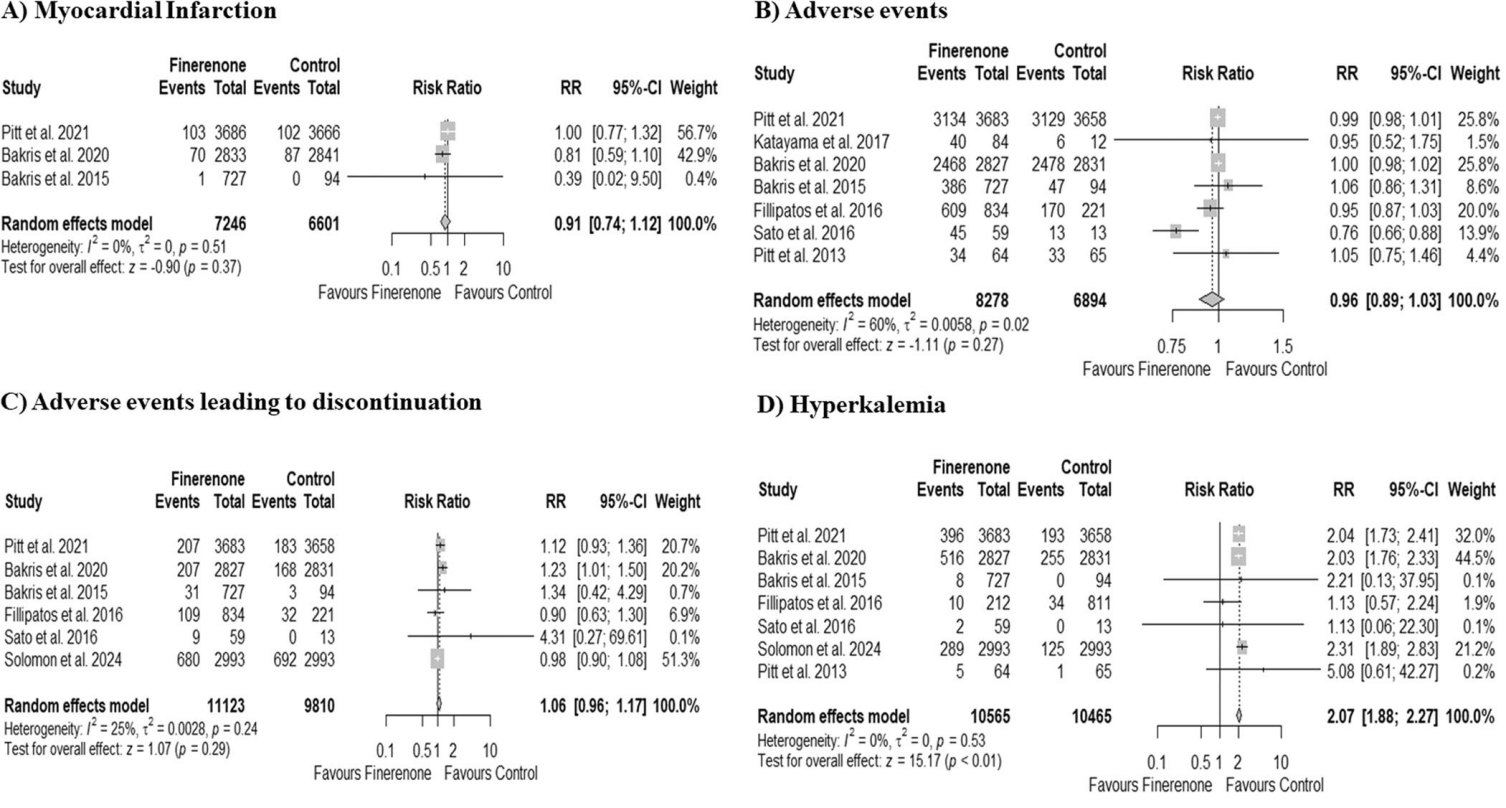
Forest plots for (A) myocardial infarction, (B) adverse events, (C) adverse events leading to discontinuation, and (D) hyperkalemia.

Three out of eight included studies reported incidence of MI. Our pooled analysis did not demonstrate a significant change in MI incidence with finerenone therapy compared to the control group (RR 0.91, 95% CI: 0.74–1.12; *p* = 0.37; Figure 4A). There was no heterogeneity between studies (*I* ^2^ = 0%).

#### 3.3.6 AEs

Seven out of eight included studies reported the rate of AEs. Our pooled analysis did not demonstrate a significant change in AEs with finerenone therapy compared to the control group (RR 0.96, 95% CI: 0.89–1.03; *p* = 0.27; Figure 4B). There was moderate heterogeneity between studies (*I* ^2^ = 60%). A sensitivity analysis was undertaken to verify the robustness of our results. The exclusion of any one study did not materially alter results or markedly reduce the demonstrated heterogeneity (Supporting Infomation S1: Figures S2–9). Six out of eight included studies reported the rate of AEs leading to discontinuation of therapy. Our pooled analysis did not demonstrate a significant change in AEs leading to discontinuation with finerenone therapy compared to the control group (RR 1.06, 95% CI: 0.96–1.17; *p* = 0.29; Figure 4C). There was low heterogeneity between studies (*I* ^2^ = 25%).

#### 3.3.7 Hyperkalemia

Seven out of eight included studies reported the incidence of hyperkalemia. Our pooled analysis demonstrated a significant increase in hyperkalemia risk with finerenone therapy compared to the control group (RR 2.07, 95% CI: 1.88–2.27; *p* < 0.01; Figure 4D). There was low heterogeneity between studies (*I* ^2^ = 26%).

## 4 Discussion

This meta‐analysis includes a total of eight RCTs involving 21 200 patients. Our study sheds light on the CV efficacy and safety of finerenone by incorporating data from the recently published FINEARTS‐HF trial [9]. The results of our pooled analysis reveal several important findings that refine our understanding of finerenone's clinical profile compared to previous meta‐analyses. Our analysis demonstrates that finerenone significantly reduces the risk of all‐cause death, HHF or unplanned hospital visits, and composite CV outcome (MACE) compared to the control group. Moreover, finerenone administration was associated with a trend of reduced CV deaths. Whereas the rates of MI, and AEs were comparable between the two groups, while the risk of hyperkalemia was increased.

By focusing on the CV outcomes in this patient population, our analysis underscores the specific advantages of finerenone in HF management and expands its clinical utility beyond renal protection. The benefits observed in HF‐related outcomes are consistent with the cardioprotective effects of MRAs [22, 23], highlighting finerenone as an effective agent in reducing the burden of HF exacerbations. The reduction in all‐cause death emphasizes finerenone's potential to improve overall survival in this high‐risk population, a finding that adds weight to its utility in routine clinical practice.

Notably, although our analysis demonstrated a trend of reduced CV death with finerenone, statistical significance was not achieved. These results are consistent with earlier studies [6], where finerenone did not demonstrate a reduction in MI or CV death. This finding suggests that while finerenone provides clear benefits in HF‐related outcomes, it may not have a substantial impact on direct coronary events. This can be explained by the fact that HF management and coronary disease require distinct therapeutic strategies [24, 25], and finerenone's primary action on the mineralocorticoid receptor may not address the underlying atherosclerotic processes that drive MI [26, 27]. Further studies, including in patients with HF with reduced ejection fraction are needed to clarify potential effects on CV death.

Additionally, our study identified a significantly higher risk of hyperkalemia in the finerenone group. This finding is consistent with the known class effects of MRAs [28]. Hyperkalemia remains a well‐recognized adverse effect, and its management is crucial to maintaining patient safety, particularly in patients with HF and chronic kidney disease [28]. However, in contrast to earlier studies, our analysis did not find significant differences in the overall incidence of adverse effects, and AEs leading to drug discontinuation between finerenone and control. These findings suggest that, despite the increased risk of hyperkalemia, finerenone is otherwise well‐tolerated, with an overall safety profile comparable to placebo.

The FINEARTS‐HF trial significantly contributes to our understanding of finerenone's efficacy. By focusing on CV outcomes, it addresses a critical gap in the literature and offers new insights into how finerenone can be used as a potential therapeutic agent in managing HF. The FINEARTS‐HF findings support the importance of finerenone in lowering CV risk, especially when it comes to HF‐related outcomes, which were not adequately addressed in earlier analyses.

Our findings also reinforce the importance of long‐term management strategies in high‐risk HF patients. The reduced risk of hospitalization or unplanned visits in the finerenone group suggests a significant impact on patient quality of life, healthcare resource utilization, and overall disease management. These benefits, when combined with a favorable safety profile, make finerenone a valuable addition to HF management. To fully understand finerenone's role in CV care, future research should concentrate on examining its long‐term CV results, particularly in different patient populations.

Some limitations should be acknowledged while interpreting our findings. We observed significant heterogeneity in the AEs outcome, which suggests potential variability in the reporting or patient populations across the included trials. Additionally, while this meta‐analysis offers valuable short‐term insights, longer follow‐up durations are needed to assess the sustained effects and safety of finerenone over time. The individual patient data meta‐analyses will provide further insights into the efficacy of finerenone as our analysis is based on study‐level data. Finally, the pooled data primarily involved patients with HF and/or kidney disease, limiting the applicability of these findings to broader populations.

## 5 Conclusion

This meta‐analysis highlights the CV efficacy of finerenone in reducing all‐cause death, HHF, and MACE. Moreover, there was no statistically significant difference between finerenone and control in terms of MI, CV death, or any AEs leading to drug discontinuation. Although a higher risk of hyperkalemia was seen with finerenone, the efficacy outweighed the potential risks. Collectively these findings support the use of finerenone in eligible patients.

## Author Contributions

conceptualization, data curation, and project administration: Mushood Ahmed, and Gregg C. Fonarow. Supervision: Gregg C. Fonarow, and Raheel Ahmed. formal analysis of data: Mushood Ahmed and Areeba Ahsan. formal analysis, methodology, and software: Mushood Ahmed, Muhammad Talha Maniya, Areeba Ahsan, and Aimen Shafiq. writing the original draft: Mushood Ahmed, Areeba Ahsan, Muhammad Talha Maniya, Javed Iqbal, and Aimen Shafiq. writing, reviewing, and editing: Raheel Ahmed, Muhammad Abdullah Naveed, Mushood Ahmed, Jamal S. Rana, Aimen Shafiq, Marat Fudim, Hritvik Jain, and Gregg C. Fonarow. visualization and validation: Mushood Ahmed, Raheel Ahmed, and Gregg C. Fonarow.

## Ethics Statement

The authors have nothing to report.

## Consent

The authors have nothing to report.

## Conflicts of Interest

Dr Fonarow reported receiving personal fees from Abbott, Amgen, AstraZeneca, Bayer, Boehringer Ingelheim, Cytokinetics, Eli Lilly, Johnson & Johnson, Medtronic, Merck, Novartis, and Pfizer outside the submitted work. Dr Fudim reported receiving personal fees from Alleviant, Ajax, Alio Health, Alleviant, Artha, Audicor, Axon Therapies, Bayer, Bodyguide, Bodyport, Boston Scientific, Broadview, Cadence, Cardioflow, Cardionomics, Coridea, CVRx, Daxor, Deerfield Catalyst, Edwards LifeSciences, Echosens, EKO, Feldschuh Foundation, Fire1, FutureCardia, Galvani, Gradient, Hatteras, HemodynamiQ, Impulse Dynamics, Intershunt, Medtronic, Merck, NIMedical, NovoNordisk, NucleusRx, NXT Biomedical, Orchestra, Pharmacosmos, PreHealth, Presidio, Procyreon, ReCor, Rockley, SCPharma, Shifamed, Splendo, Summacor, SyMap, Verily, Vironix, Viscardia, and Zoll; and receiving grants from the National Institutes of Health, Doris Duke, outside the submitted work. No other disclosures were reported.

## Supporting information

Supporting information.

## Data Availability

All data generated or analyzed during this study are included in this article. Further inquiries can be directed to the corresponding author.
