# Supplementary material for: Cardiovascular Efficacy and Safety of Finerenone: A Meta‐Analysis of Randomized Controlled Trials
Source: Clin Cardiol. 2025 Feb 6;48(2):e70065. doi: 10.1002/clc.70065 (PMC11799767; doi:10.1002/clc.70065)

**Supplementary Appendix**

Table S1: Detailed search strings used for major databases/Register

| Database/Register | Search string | Records |
| --- | --- | --- |
| PubMed/MEDLINE | ("finerenone"[All Fields] OR "BAY 94-8862"[All Fields] OR "MR antagonist"[All Fields] OR "non-steroidal mineralocorticoid receptor antagonist"[All Fields]) AND ("heart failure"[All Fields] OR "cardiac failure"[All Fields] OR "congestive heart failure"[All Fields] OR "CHF"[All Fields] OR "HFrEF"[All Fields] OR "HFpEF"[All Fields] OR "chronic kidney disease"[All Fields] OR "CKD"[All Fields] OR "kidney failure"[All Fields] OR "renal insufficiency"[All Fields] OR "nephropathy"[All Fields] OR "type 2 diabetes"[All Fields] OR "T2DM"[All Fields] OR "diabetes mellitus"[All Fields] OR "hyperglycemia"[All Fields]) | 554 |
| Embase | ('finerenone'/exp OR 'finerenone' OR 'bay 94-8862' OR 'mr antagonist' OR 'non-steroidal mineralocorticoid receptor antagonist') AND ('heart failure'/exp OR 'cardiac failure' OR 'congestive heart failure' OR 'chf' OR 'hfref' OR 'hfpef' OR 'chronic kidney disease'/exp OR 'ckd' OR 'kidney failure' OR 'renal insufficiency' OR 'nephropathy' OR 'type 2 diabetes mellitus'/exp OR 'type 2 diabetes' OR 't2dm' OR 'diabetes mellitus' OR 'hyperglycemia') | 1197 |
| Cochrane CENTRAL | (("finerenone" OR "BAY 94-8862" OR "MR antagonist" OR "non-steroidal mineralocorticoid receptor antagonist") AND ("heart failure" OR "cardiac failure" OR "congestive heart failure" OR "CHF" OR "HFrEF" OR "HFpEF" OR "chronic kidney disease" OR "CKD" OR "kidney failure" OR "renal insufficiency" OR "nephropathy" OR "type 2 diabetes" OR "T2DM" OR "diabetes mellitus" OR "hyperglycemia")) | 216 |
| ClinicalTrials.gov | finerenone  AND  ("heart failure" OR "congestive heart failure" OR "CHF" OR "HFrEF" OR "HFpEF"  OR  "chronic kidney disease" OR "CKD" OR "kidney failure" OR "renal insufficiency" OR "nephropathy"  OR  "type 2 diabetes" OR "T2DM" OR "diabetes mellitus" OR "hyperglycemia") | 37 |

Figure S1: PRISMA flowchart showing the study selection process


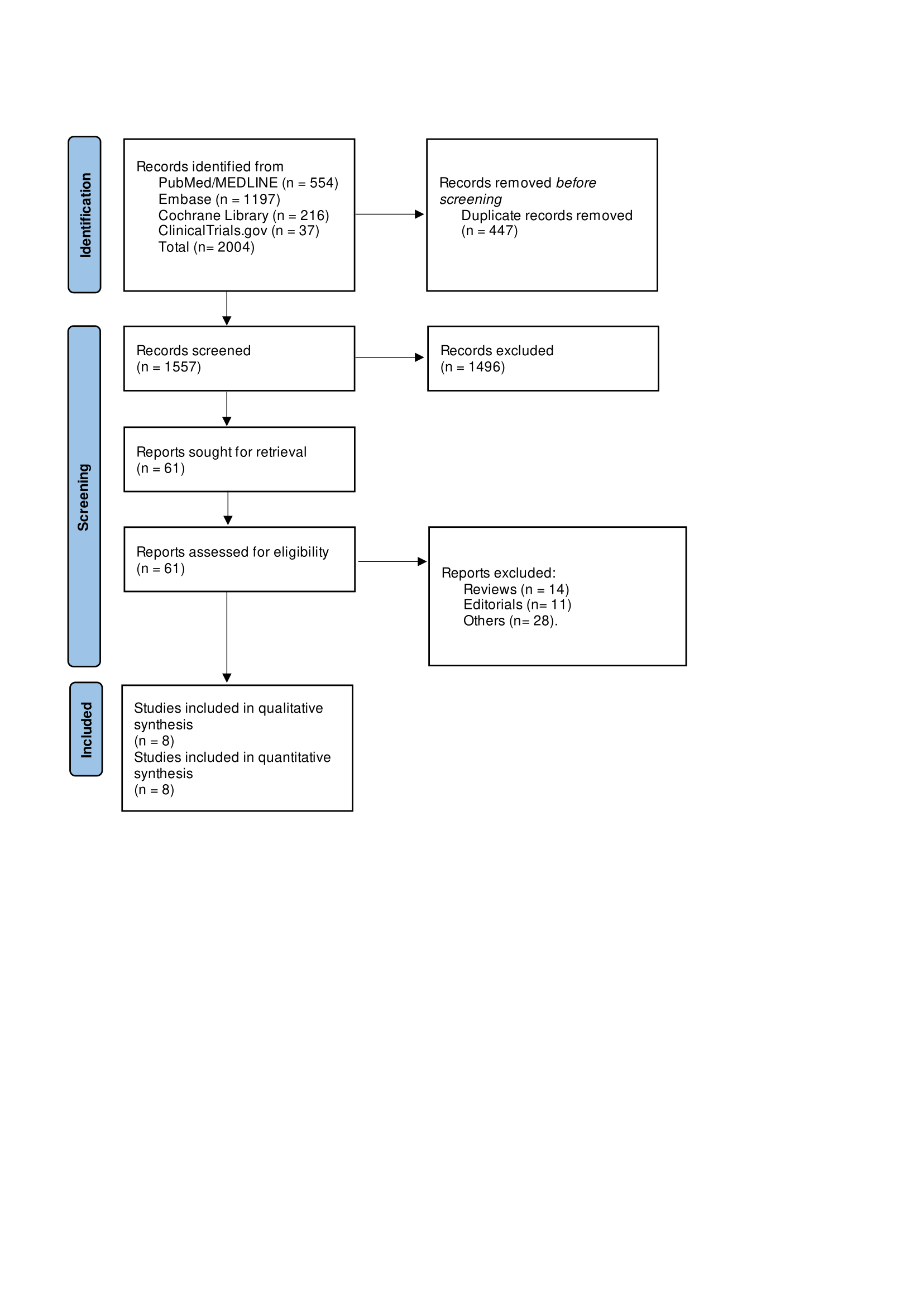


Figure S2: Leave-one-out sensitivity analysis for all-cause death


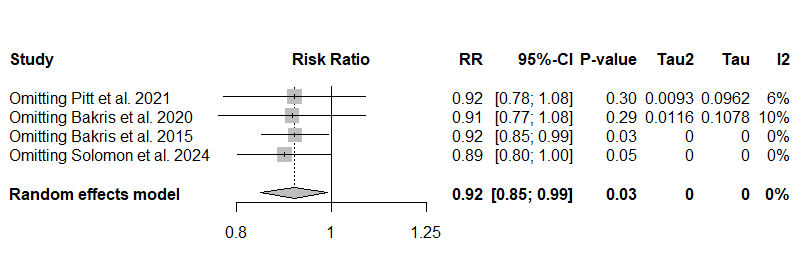


Figure S3: Leave-one-out sensitivity analysis for cardiovascular death


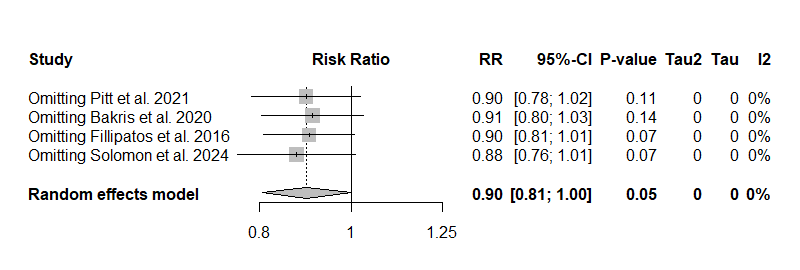


Figure S4: Leave-one-out sensitivity analysis for MACE


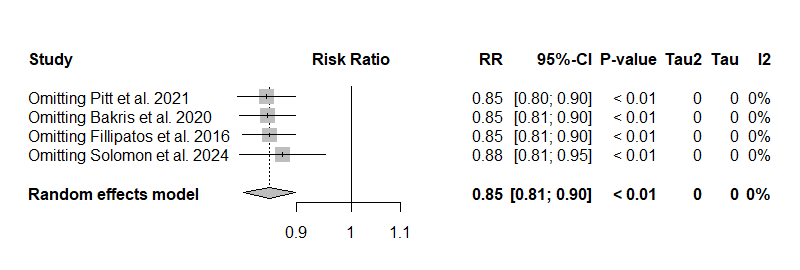


Figure S5: Leave-one-out sensitivity analysis for Heart Failure-Related Hospitalizations or unplanned visits


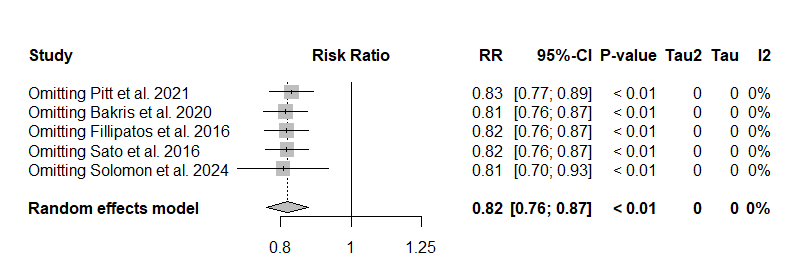


Figure S6: Leave-one-out sensitivity analysis for Myocardial Infarction


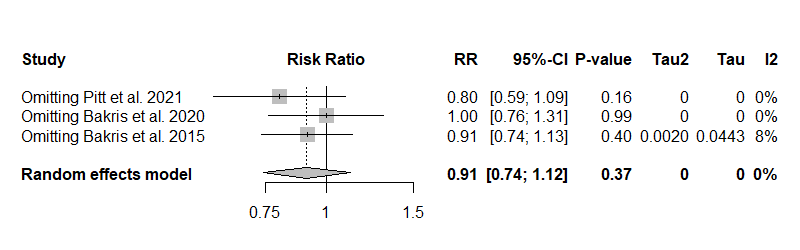


Figure S7: Leave-one-out sensitivity analysis for adverse events


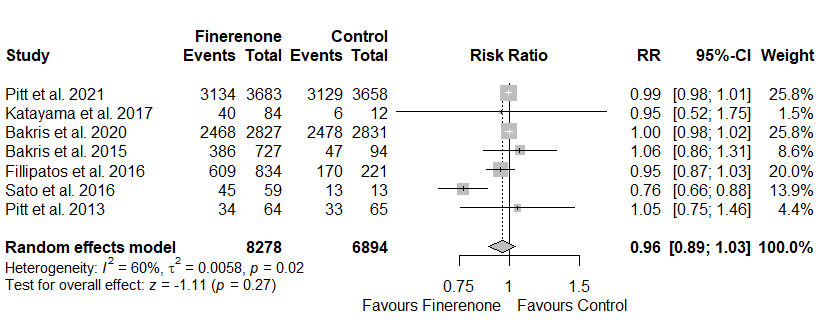


Figure S8: Leave-one-out sensitivity analysis for adverse events leading to discontinuation


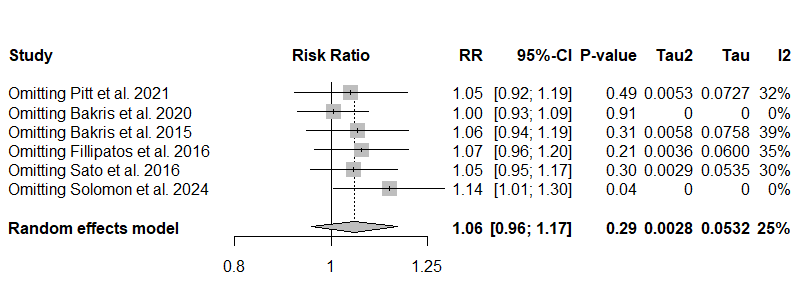


Figure S9: Leave-one-out sensitivity analysis for hyperkalemia


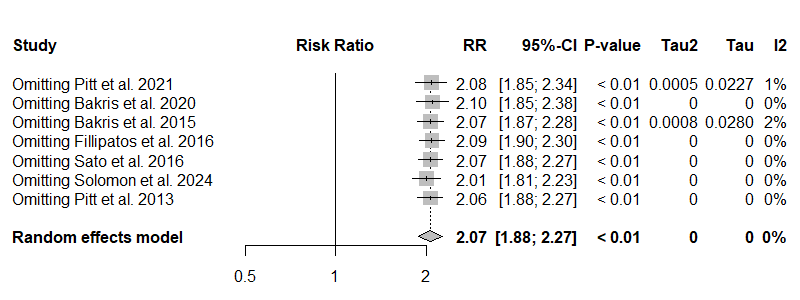

Supplement: Supplementary file 1 — Supporting information. [file CLC-48-e70065-s001.docx]
